# Evaluation of Antimicrobial Effectiveness of Lemongrass, Ash Gourd, Watermelon, Guava Extracts, Chlorhexidine Against Streptococcus mutans: An In Vitro Study

**DOI:** 10.7759/cureus.76948

**Published:** 2025-01-05

**Authors:** Gauri Nair, Rhujuta Mokal, Amit Patil, Minakshi Bhattacharjee, Sheetal Mali, Ashwini S Panchmahalkar, Sanpreet S Sachdev

**Affiliations:** 1 Conservative Dentistry and Endodontics, Bharati Vidyapeeth (Deemed to be University) Dental College and Hospital, Navi Mumbai, Mumbai, IND; 2 General Microbiology, Bharati Vidyapeeth (Deemed to be University) Dental College and Hospital, Navi Mumbai, Mumbai, IND; 3 Oral Pathology and Microbiology, Bharati Vidyapeeth (Deemed to be University) Dental College and Hospital, Navi Mumbai, Mumbai, IND

**Keywords:** anti-bacterial action, anti-inflammatory action, dental caries, streptococcus mutans, zone of inhibition

## Abstract

Introduction

The oral cavity holds a vital role, as it harbors diverse microbial communities, with *Streptococcus mutans* being a major contributor to dental caries. While antimicrobial agents such as chlorhexidine target oral bacteria, their use may lead to genotoxic effects, highlighting the need for safer alternatives. This study investigates the antimicrobial properties of lemongrass, ash gourd, watermelon, and guava against *S. mutans*.

Materials and methodology

Extracts of lemongrass, ash gourd, watermelon, and guava were evaluated using the well diffusion method, measuring the zone of inhibition around each well. Each extract was tested in triplicate for effective individual assessment of results.

Results

Lemongrass extract demonstrated the highest zone of inhibition at 28 mm, followed by guava extract at 22 mm. In contrast, watermelon and ash gourd extracts showed no inhibitory activity.

Conclusion

Lemongrass exhibits the strongest antimicrobial efficacy against *S. mutans*, with guava as a secondary option, while watermelon and ash gourd were ineffective. Further clinical trials are recommended to explore lemongrass and guava as potential antimicrobial agents in oral health management.

## Introduction

The oral cavity hosts a diverse range of microbial communities that include bacteria, fungi, viruses, and protozoa. These are collectively known as the oral microbiota. The shift in the equilibrium of these microbial communities results due to the release of secondary metabolites can give rise to a number of dental issues such as dental caries and periodontal diseases. Dental caries is a most common chronic disease that occurs because of acid-producing microorganisms, dietary carbohydrates, and host characteristics. This process begins with the microbial plaque and thus forms a biofilm. It results in the demineralization of inorganic substances and thus causes the breakdown of tooth structure [1]. *Streptococcus mutans* is a non-motile, gram-positive cocci that metabolizes carbohydrates. It is a facultative anaerobe that plays a crucial role in this process and acts as a primary contributor to dental caries [2].

Furthermore, our oral cavity, with complex surfaces and conditions, provides an intricate environment for these microbes to thrive. Thus, meticulous cleaning becomes a challenge that extends to every domain of oral hygiene maintenance, including patients with intraoral appliances [3]. To combat these shortcomings, the use of mouthwashes and disinfectant solutions grew in favor. Mouthwashes in dentistry serve preventative and therapeutic roles through chemical and mechanical mechanisms [4,5]. Chlorhexidine is the most effective agent against *S. mutans* and plaque. It acts as a strong antiseptic that disrupts bacterial membranes and inhibits plaque formation. 

Chlorhexidine, when used in clinical applications such as mouthwashes or topical gels, has been associated with a variety of side effects. The most common include dry mouth (xerostomia), changes in taste perception, particularly affecting salt and bitter flavors, and discoloration or a coated tongue. Some studies have also reported that the use of 0.12% chlorhexidine can lead to an increase in plaque formation in teeth [6]. Other frequently observed adverse effects include oral paresthesia, enlargement of the parotid glands, desquamation of the oral mucosa, and a burning sensation in the mouth [7].

One of the most undesirable effects of chlorhexidine is dental staining, which results from the formation of colored metal sulfides in the pellicle and non-enzymatic browning (the Maillard reaction) [8-10]. More severe adverse reactions include hypersensitivity, with risks of both type I and type IV reactions, potentially leading to anaphylaxis. The incidence of such reactions is reported to be 0.78 per 100,000 exposures [11]. Case studies have highlighted instances where chlorhexidine mouthwash caused severe allergic reactions, including respiratory arrest and even death [12]. A growing concern is the development of antimicrobial resistance, wherein microorganisms targeted by chlorhexidine adapt and become resistant, diminishing the effectiveness of the product [13].

This research, thus, focuses on a specific herbal mouthwash that has shown favorable antimicrobial effects in previous investigations. The plant-based ingredients such as tannins, alkaloids, and flavonoids are scientifically proven to have antimicrobial properties. Traditional medicine manuals often suggest the use of plant-based treatments for managing various infections. These plant-based products have easy availability, fewer side effects, and lower toxicity than synthetic medications [14]. Thus, the present study used lemongrass, ash gourd, watermelon, and guava extracts to compare their antimicrobial efficacy against *S. mutans*.

Lemongrass (*Cymbopogan Citratus*) contains the essential compound citral and other bioactive compounds such as alkaloids, flavonoids, and phenolic compounds with tannins, thus contributing to the antibacterial properties of lemongrass [15]. The extracts from the seeds and pulp of ash gourd (*Benincasa hispida*) have been tested against various periodontal pathogens. They have demonstrated the ability to suppress microbial growth [16]. Watermelons (*Citrullus lanatus*) are used for their pharmacological properties, including antibacterial, antifungal, antimicrobial, and anti-inflammatory effects [17]. The guava leaves (*Psidium guajava L.*) have extensive antibacterial properties and treat various ailments. Guava inhibits bacteria, wherein the flavonoids in the leaves, such as quercetin and guaiaverin, are responsible for this antibacterial effect. These compounds from guava leaves provide antibacterial activity against *S. mutans* [18].

Previous studies have explored the antibacterial properties of various fruits against a wide range of pathogens, demonstrating their potential as natural antimicrobial agents and their ability to be used in oral healthcare. However, these studies have limited emphasis on *S. mutans*, a major etiological agent in dental caries [19,20]. While several phytochemical investigations of lemongrass, ash gourd, watermelon, and guava have indicated general antibacterial activity [21], the specific efficacy of these fruits and leaves against *S. mutans* remains largely unexplored. The current study is distinctive in its focus on evaluating the antibacterial effects of lemongrass, ash gourd, guava, and watermelon, specifically against *S. mutans*. This approach provides novel insights into the potential of these fruits as targeted antimicrobial agents for the prevention and management of dental caries, distinguishing our research from previous studies in this domain. Additionally, the long-term side effects associated with the use of chlorhexidine, such as tooth staining and altered taste, can be avoided with these herbal extracts, as they show no significant side effects. These extracts can potentially be incorporated into mouthwashes and dentifrices, offering an alternative that harnesses their antibacterial properties while minimizing adverse effects [22].

The urge for this study is the lack of literature comparing the antimicrobial activity of lemongrass, ash gourd, watermelon, and guava extracts against *S. mutans*. Even though these extracts are readily available, economical, and possess medicinal properties, chlorhexidine will remain the gold standard antimicrobial agent for comparison. Therefore, the present research aims to evaluate the efficacy of these four plant extracts as a potent antimicrobial agent against *S. mutans*.

## Materials and methods

The present in vitro study was conducted over a period of three months, from March to May 2023. The Institutional Review Board permitted the ethical conduct and protocol of the study. (Protocol No.: IEC358072022, dated 12/07/2022)

Bacterial strains and culture conditions

*S. mutans* ATCC 25175 culture was subcultured in a blood agar medium with 5-10% CO_2_ and was anaerobically incubated at 37℃ for 48 hours.

Preparation of the ethanolic extracts

**Figure 1:**
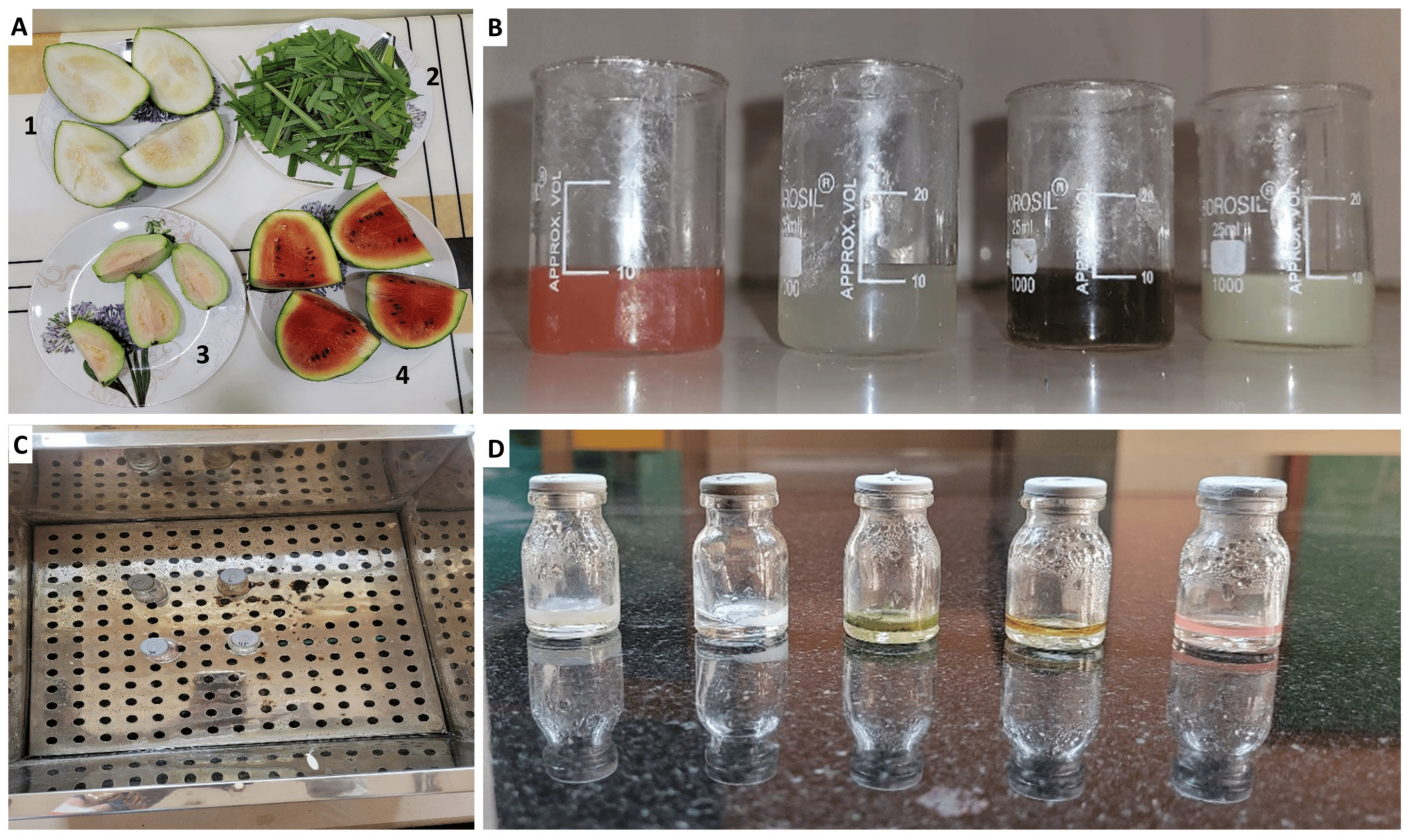
A) Chopped pieces of ash gourd (1), lemongrass (2), guava (3), and watermelon (4). B) Ethanolic extracts of the fruits and leaves. C) Filtrates kept in water bath for evaporation of the solvent. D) Final extracts of all the fruits used for antimicrobial sensitivity analysis.

The leaves and fruits of lemongrass, ash gourd, watermelon, and guava were washed under tap water for 2 minutes, following which they were chopped into pieces of uniform sizes. The seeds of the fruits were removed. The chopped portions were then sundried in the open air for 10 days. After sufficient drying, the samples were ground into a coarse powder using mortar and pestle, dried again in the oven at 45°C for 3 days, and then micronized into a fine powder using an electric blender. All the samples were kept in clean, closed glass containers and stored until use. 10 gm of each respective material was weighed on an electrical weighing machine. The powder was dissolved in 100 mL of 95% ethanol and incubated at 35°C for 60 minutes at 120 rpm. The mixture was filtered through a 0.45 µm membrane filter, and the filtrate was kept in a water bath at 60°C to allow the evaporation of the solvent (Figure 1). Until it was used, the extracted material was stored at 4°C away from sunlight in a refrigerator [23]. For the microbial sensitivity analysis, 2% chlorhexidine was used as a positive control (group I), and distilled water was used as a negative control (group II). The other groups included lemongrass (group III), ash gourd (group IV), watermelon (group V), and guava (group VI). 

Antimicrobial susceptibility test

The agar well diffusion method was used for its antibacterial effectiveness to test the ethanolic extracts of lemongrass, ash gourd, watermelon, and guava. The blood agar medium was suspended in distilled water and autoclaved at 121°C with 15 lb/sq inch pressure for 20 minutes. A seven-day-old culture of the test organism was spread evenly on a plate after inoculation in peptone water with an incubation period of four hours. Subsequently, three wells with an 8 mm diameter each were carefully formed in the blood agar plate using a sterile cork borer. Thus, each well was treated with a mixture of lemongrass, ash gourd, watermelon, and guava against 2% chlorhexidine. The inoculated sample plates were then incubated at 37°C for 48 hours.

Outcome assessment

The primary outcome examined was the mean zone of inhibition. It was measured as the diameter in millimeters of the zone formed around the well of the respective material. Secondary outcomes comprised the antimicrobial activity of the two concentrations of the materials (25% and 50%).

## Results

**Figure 2:**
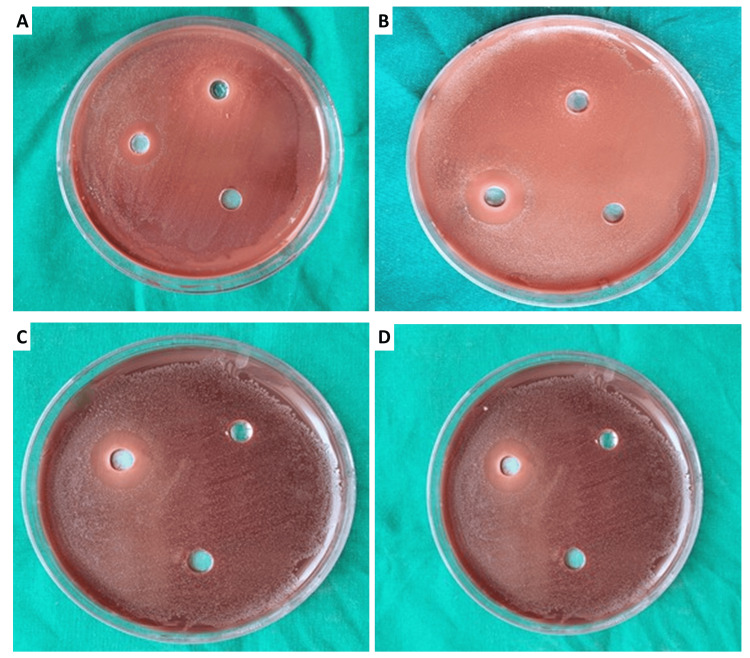
(A) Lemongrass extract exhibited the highest zone of inhibition against Streptococcus mutans. (B) Guava extract exhibited the lowest zone of inhibition against S. mutans. (C) Ash gourd extract showed no antimicrobial activity. (D) Watermelon extract showed no antimicrobial activity.

All extracts were individually evaluated using a well diffusion method against *S. mutans*. *S. mutans* was seen at different concentrations (25% and 50%) of the extracts for a 48-hour incubation period. At 25% concentration, all the extracts showed no antimicrobial activity. At 50% concentration, the below stated are the results:

The highest zone of inhibition was shown by lemongrass against *S. mutans*, followed by guava extract. No zone of inhibition was seen in the ash gourd and watermelon extracts (Figure 2). The results for each extract were noted at the end of 48 hours (Table 1).

**Table 1:** Zone of inhibition under different concentrations

Extracts	Zone of inhibition under different concentrations (in mm)	
	25%	50%
Lemongrass	0 mm	28 mm
Guava	0 mm	22 mm
Ash gourd	0 mm	0 mm
Watermelon	0 mm	0 mm

A statistically highly significant (p<0.01) difference was observed in the inhibition zones between 25% and 50% concentrations of lemongrass and a similar difference between the two concentrations of guava. The ANOVA test also revealed a statistically highly significant difference (p<0.01) between the inhibition zones achieved by the different materials. To further analyze these differences, a pairwise comparison was performed, which showed that lemongrass and guava both showed significantly greater zones of inhibition (p<0.01) than ash gourd and watermelon; however, there was no statistically significant difference (p>0.05) between the zones of inhibition achieved by the former two materials. Likewise, there was a statistically non-significant difference between ash gourd and watermelon, both of which were unable to achieve any zone of inhibition.

## Discussion

Dental caries, the second most common disease after the common cold, is primarily caused by *S. mutans*. Chlorhexidine, a broad-spectrum antimicrobial considered the standard for plaque control in dentistry, has side effects such as altered taste, tooth staining, and calculus formation. Due to these limitations, researchers are exploring traditional medicine for new antimicrobial agents, particularly from plant sources. Many medicinal plant compounds have shown effectiveness in preventing dental biofilms [24]. Hence, this study emphasizes the potential of herbal extracts with fewer side effects than synthetic drugs. Thus, more in vitro studies and clinical trials should be conducted to identify effective natural alternatives to synthetic products, especially considering the accessibility and safety issues associated with conventional mouthwashes such as chlorhexidine [25]. Hence, ethanolic extracts of lemongrass, guava, watermelon, and ash gourd were used to determine antimicrobial efficacy against *S. mutans*. Well diffusion method was used to evaluate the efficacy of each extract against *S. mutans*. *S. mutans* were exposed to varying concentrations (25% and 50%) during a 48-hour incubation period in different ratios (1:4 and 1:1) of the extracts.

 Lemongrass exhibits antimicrobial activity against multiple microbes at ≤2% concentrations. Its antioxidant effects stem from citral (neral and geranial) and citronellal constituents [26]. Different species of lemongrass have been used because of their medicinal properties and beneficial impact on health. They have digestive, antioxidant, antimicrobial, anti-inflammatory, anti-carcinogenic, and antimutagenic properties [27]. Our study shows that lemongrass has the greatest zone of inhibition at 28 mm, whereas a previous study showed a 10.7 mm zone of inhibition [28]. The present study shows lemongrass extract possesses a promising antimicrobial activity against *S. mutans*. Extensive research has focused on guava leaves because of their antimicrobial properties and versatile uses in treating various diseases. Our present study shows that guava extract shows a zone of inhibition of 22 mm, whereas a study showed an 18 mm zone of inhibition [29].

The watermelon contains various bioactive compounds, and its peel has high levels of saponins and flavonoids, contributing to antimicrobial activity, which is caused by disrupting microbial membranes and cell walls. The tannins inhibit enzyme activity and protein synthesis, thus damaging the microbial structures and affecting biofilm formation. These findings suggest that the phytochemical composition of these plant extracts, particularly the saponins, flavonoids, terpenoids, and tannins, contribute to their antimicrobial and anti-biofilm properties. It could be used for potential therapeutic applications. It was reported that the zone of inhibition obtained by watermelon against *S. mutans* was 10 mm [30]. However, our current study found no zone of inhibition against *S. mutans*.

Different parts of the ash gourd plant, including peel, which has better antioxidant properties, and the pulp and seed, which have better antimicrobial and antifungal properties, are widely used in Indian traditional medicine to treat various health issues due to their diverse therapeutic properties, including anti-inflammatory and antioxidant effects [31]. Due to the lack of literature on the antimicrobial efficacy of ash gourd against *S. mutans*, the present study aimed to detect its efficacy against the* *bacterium. According to the findings of the present study, we can infer that ash gourd shows no zone of inhibition against *S. mutans*.

The observations from the present study can also serve as an initial step for further phytochemical studies on these plants to isolate active compounds and evaluate their effectiveness against various bacterial strains and dental caries. There should be future investigations that evaluate the safety and efficacy of these plant-based compounds as new therapeutic options for infectious diseases. It should explore their potential to develop antibacterial mouth rinses and oral preparations.

While still less effective than chlorhexidine, plant extracts showed effective antimicrobial activity against oral bacteria in vitro, highlighting their potential as natural alternatives for patients seeking to avoid alcohol and artificial additives. Even though there is less literature available on the use of naturopathic extracts in the form of mouthwashes, more such in vitro studies should be indicated for further investigation. Nevertheless, more clinical application of these extracts is required to recognize the enormous ability of these extracts in caries control. Thus, we can conclude that in dental treatment, naturopathies can be an alternative to synthetic antimicrobial medications since they have fewer side effects. As these natural extracts with anti-inflammatory and antibacterial qualities are ideal for adding to mouthwashes and oral sprays, they can be used at their full potential as an alternative to chlorhexidine in clinical practices.

Limitations of the study

The present study was conducted in an in vitro setting with controlled laboratory conditions. This setup does not replicate a real-life oral cavity scenario wherein a complex environment may affect the clinical performance of the materials. The oral cavity contains saliva, pH modifiers, biofilms, and individual variations that could influence the antimicrobial activity of these substances. Additionally, only a single microorganism, *S. mutans*, which is the primary cariogenic organism, was considered, while the dental and periodontal diseases have a polymicrobial etiology. The extracts were tested at only two concentrations (25% and 50%). A broader range of concentrations could provide more insights into the minimum inhibitory concentration and optimal effective dose. Overall, the study provides results only limited to the studied concentrations of 25% and 50% of the materials on *S. mutans*. Future studies can assess the effects of varying concentrations of the respective materials used in the study.

## Conclusions

Naturopathic remedies offer a superior alternative for addressing oral and dental health concerns compared to synthetic antimicrobial medications. These remedies can minimize adverse effects on the body and effectively combat issues related to side effects. The present study demonstrated the remarkable efficacy of lemongrass and guava extracts in combating cariogenic bacteria, such as *S. mutans*. These natural extracts, rich in bioactive compounds, possess potent antibacterial, anti-inflammatory, and antimicrobial properties, making them ideal for incorporation into mouthwashes and oral sprays. Nevertheless, further in vivo research and clinical trials are imperative to validate and reinforce the utilization of these natural agents.

## Figures and Tables

**Figure 3:**
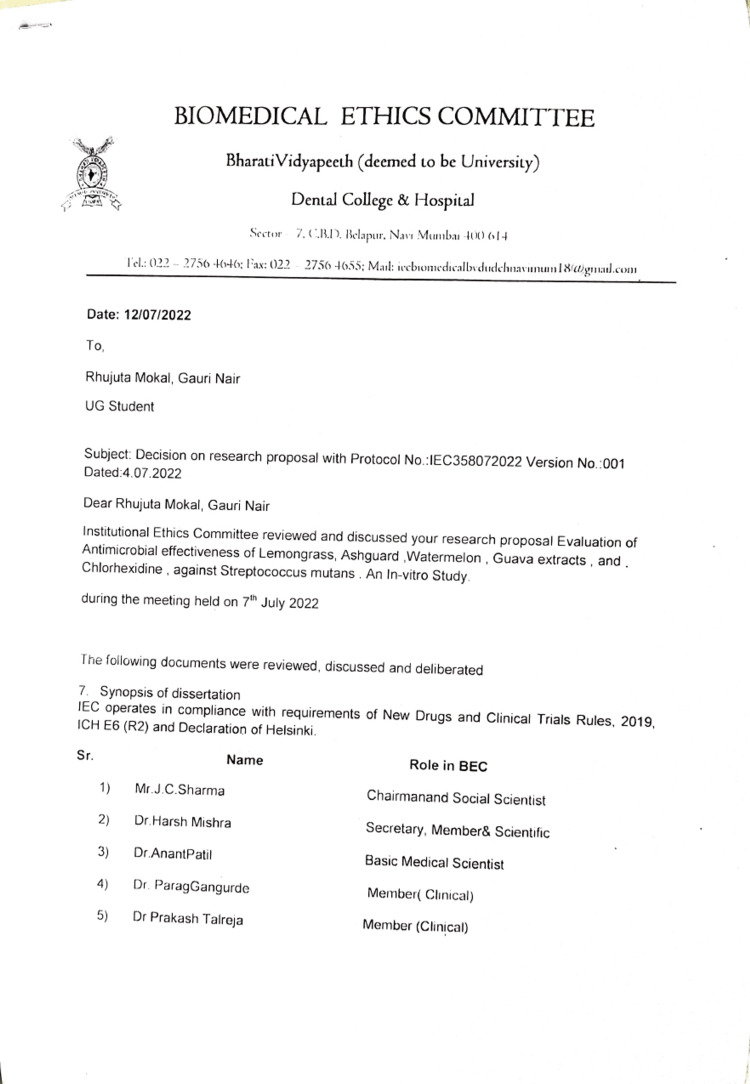
Ethical approval letter first page

**Figure 4:**
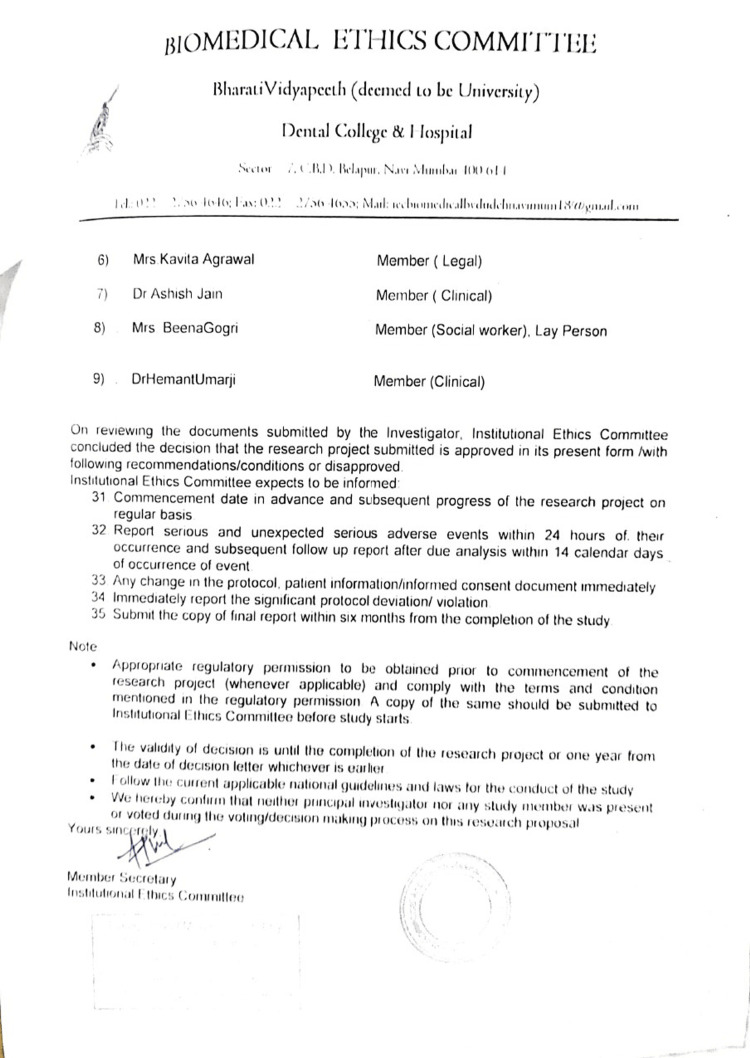
Ethical approval letter second page
